# Versatile Physiological Functions of Plant GSK3-Like Kinases

**DOI:** 10.3390/genes12050697

**Published:** 2021-05-08

**Authors:** Juan Mao, Wenxin Li, Jing Liu, Jianming Li

**Affiliations:** 1 State Key Laboratory for Conservation and Utilization of Subtropical Agro-Bioresources, College of Forestry and Landscape Architecture, South China Agricultural University, Guangzhou 510642, China; liwenxin@stu.scau.edu.cn (W.L.); liujing3925@126.com (J.L.); 2 Guangdong Key Laboratory for Innovative Development and Utilization of Forest Plant Germplasm, College of Forestry and Landscape Architecture, South China Agricultural University, Guangzhou 510642, China; 3 Department of Molecular, Cellular, and Developmental Biology, University of Michigan, Ann Arbor, MI 48109, USA

**Keywords:** brassinosteroid signaling, protein phosphorylation, biotic/abiotic stress, stomata development, flowering, cell division/differentiation, cell expansion/elongation, root development, vascular differentiation, photomorphogenesis

## Abstract

The plant glycogen synthase kinase 3 (GSK3)-like kinases are highly conserved protein serine/threonine kinases that are grouped into four subfamilies. Similar to their mammalian homologs, these kinases are constitutively active under normal growth conditions but become inactivated in response to diverse developmental and environmental signals. Since their initial discoveries in the early 1990s, many biochemical and genetic studies were performed to investigate their physiological functions in various plant species. These studies have demonstrated that the plant GSK3-like kinases are multifunctional kinases involved not only in a wide variety of plant growth and developmental processes but also in diverse plant stress responses. Here we summarize our current understanding of the versatile physiological functions of the plant GSK3-like kinases along with their confirmed and potential substrates.

## 1. Introduction

Glycogen synthase kinase 3 (GSK3)-like kinases, also known as SHAGGY-like kinases that were named after the morphological phenotype of a *Drosophila melanogaster* GSK3-defective mutant [1], are a class of highly conserved multifunctional protein serine/threonine kinases in all eukaryotes. These kinases are constitutively active under normal cellular conditions but become inactivated in response to a wide range of developmental cues and environmental signals. In mammals, there are only two structurally similar GSK3 isoforms, GSK3α and GSK3β, which regulate diverse biochemical and cellular processes, such as glycogen metabolism, cell division and differentiation, and oncogenesis, with over 100 known substrates [2,3,4,5,6]. In plants, GSK3-like kinases are encoded by a multigene family (10 in Arabidopsis and 9 in rice) consisting of four subfamilies (Table 1) [7,8]. Since their initial discoveries in 1993 [9,10], many molecular and genetic studies have shown that the plant GSK3-like kinases are also multitasking kinases involved in a wide range of developmental and stress response processes by binding and phosphorylating diverse protein substrates, including receptors, kinases, ubiquitin ligases, metabolic enzymes, cyclins, and transcription factors [11]. In this review, we summarize major cellular and physiological functions of the plant GSK3-like kinases from studies performed in many model and non-model plant species.

## 2. A Key Regulator of Brassinosteroid Signaling 

In Arabidopsis, ten AtSKs (*Arabidopsis thaliana* Shaggy/GSK3-like Kinases) are classified into four subgroups (from I to IV) [7] (Table 1). At least seven of them, including all three members of the subgroup II, AtSK21/BIN2 (BRASSINOSTEROID-INSENSITIVE2), AtSK22/BIL2 (BIN2-LIKE2), and AtSK23/BIL1, are implicated in regulating the intracellular signal transduction of the plant steroid hormones [12,13,14,15,16,17], brassinosteroids (BRs) that are essential for plant growth, development, and stress tolerance [18]. BIN2, the first plant GSK3-like kinase genetically discovered by altered plant morphology and BR insensitivity [19,20,21,22], was found to be a key negative regulator of the BR response in Arabidopsis. BIN2 interacts and phosphorylates BZR1 (Brassinazole-Resistant1) and BES1 (bri1-EMS Suppressor1) [23,24], which are two key transcriptional factors regulating thousands of BR-responsive genes [25,26,27,28]. Such BIN2-catalyzed BZR1/BES1 phosphorylation decreases their protein stability, retains them in the cytosol via phosphorylation-dependent interactions with 14-3-3 proteins, and/or directly inhibits their DNA binding activity. In Arabidopsis roots, BIN2 phosphorylates and stabilizes PUB40 (Plant U-Box40), an Arabidopsis U-box-containing E3 ubiquitin ligase to enhance the PUB40-BZR1 interaction, thus promoting BZR1 degradation and reducing BR signaling [29]. Recent studies have also shown that BIN2 phosphorylates an Arabidopsis MYB (myeloblastosis) protein MYBL2 (MYB-Like2) and HAT1 (*Arabidopsis thaliana* homeodomain-leucine zipper protein1) to stabilize their protein stability, thereby assisting BES1 to downregulate BR-repressed genes [30,31]. Several members of BSKs (BR Signaling Kinases), which are implicated in transmitting the extracellular BR signal into the cytosol [32,33], including BSK1, BSK3, BSK5, BSK6, BSK8, and BSK11, physically interact with and are phosphorylated by BIN2 and AtSK22/BIL2 [33]. It was shown that the BIN2-catalyzed phosphorylation of BSK3 promotes the interactions of BSK3 with the BR receptor BRI1 (Brassinosteroid-Insensitive1) and a protein serine/threonine phosphatase BSU1 (Bri1 Suppressor1) to enhance BR signaling [34]. Thus, BIN2 likely has a dual function in regulating BR signaling by promoting the BSK3-BSU1 interaction while inhibiting the signaling activities of the two key transcriptional factors BES1 and BZR1. However, it remains to be investigated to fully appreciate the opposite impact of BIN2 on BR signaling given an earlier evolution and genetic study that questioned the role of BSU1, which is expressed specifically in pollen and present only in the Brassicaceae family, in regulating BR signaling [35]. 

In rice, several members of OsSKs (*Oryza sativa* Shaggy/GSK3-like Kinases), including OsSK22/GSK2, also play key negative roles in BR signaling. It was shown that OsSKs phosphorylate and inactivate various transcription factors known to regulate BR responses in rice, including OsBZR1 (*Oryza sativa* BZR1 homolog), DLT (Dwarf and Low-Tillering), LIC (Leaf and tiller angle Increased Controller), RLA1 (Reduced Leaf Angle1)/SMOS1 (Small Organ Size1), OFP3 (Ovate Family Protein3) and OFP8 [36,37,38,39,40,41] (Table 1). It is interesting to note that all these transcriptional factors, except LIC and OFP3, function as positive regulators of the rice BR signaling. Similar to what was discovered in Arabidopsis [29], OsSK22/GSK2 can also phosphorylate and stabilize a rice PUB-containing E3 ubiquitin ligase, OsPUB24, to promote the proteasome-mediated degradation of OsBZR1 [42], thus reducing the BR signaling output. It is important to note that the nomenclature of the rice GSK3-like kinases is quite confusing in the literature, especially for many of the studies that used “OsGSK2” to name the rice GSK2 (OsSK22) that was widely considered as the rice ortholog of BIN2.

## 3. Roles in Plant Growth and Development

In addition to their critical roles in regulating the intracellular transmission of the extracellular BR signals, the plant GSK3-like kinases are also known to be involved in several other plant growth and development processes, such as stomatal development, root growth, vascular differentiation, and flower development, through regulating the protein stability, subcellular localizations, and/or biochemical activities of additional transcriptional factors and signaling proteins. In this section, we mainly discuss the regulatory mechanisms by which plant GSK3-like kinases biochemically interact with those transcription factors and signaling components to execute their physiological functions in various plant growth and developmental processes. 

### 3.1. Regulating Cell Division and Cell Elongation/Expansion

**Figure 1:**
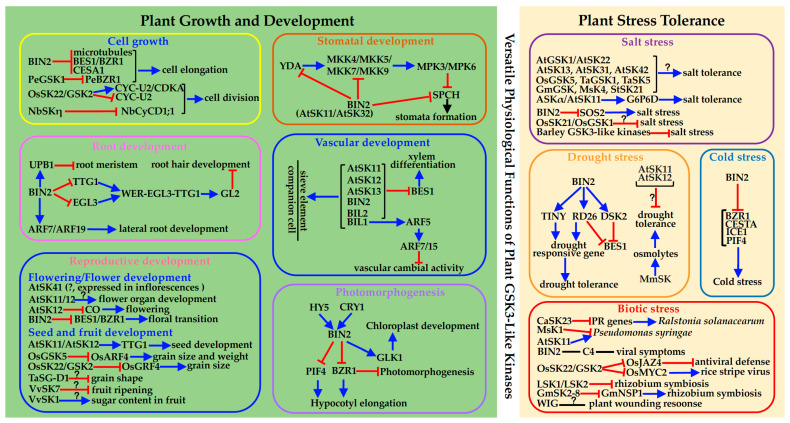
A graphic summary of known physiological functions of plant GSK3-like kinases. The blue → and red ⊣ indicate stimulatory and inhibitory effects/interactions between indicated proteins and their interacting proteins or listed developmental/physiological processes, respectively. The question marks denote unknown mechanisms by which indicated GSK3-like kinases influence plant physiological/developmental processes.

Cell division, cell expansion/elongation, and cell differentiation are fundamental to plant growth and development. One of the original studies that implicated the plant GSK3-like kinases in BR signaling revealed greatly reduced cell size of leaves and hypocotyls in gain-of-function *ultracurvata1* (*ucu1*) mutants (allelic to *bin2*) that had similar numbers of cells compared to their wild-type control [22]. Consistent with the functional redundancy between the Arabidopsis GSK3-like kinases, an earlier study showed that overexpression of a mutant variant of AtSK32 (AtSK32-Arg^178^Ala), greatly reduced the cell size in floral organs. Similarly, a *Phyllostachys edulis* GSK3-like kinase 1 (PeGSK1) was recently shown to interact with PeBZR1 (a Moso bamboo homolog of the Arabidopsis BZR1) and to function as a negative regulator of cell growth [43]. Over-expression of PeGSK1 in Arabidopsis markedly inhibited plant growth and greatly reduced expression of many BZR1-target genes involved in cell elongation, leading to many characteristic phenotypes of BR-deficient/signaling mutants such as reduced cell sizes, smaller leaves and a dwarfed statue (Figure 1) [43]. In addition to its indirect impact on cellular growth through regulating the stability, subcellular locations, and DNA-binding activities of BES1/BZR1, BIN2 interacts with tubulins and microtubules to directly control cell expansion, thus regulating pavement cell formation [44]. Consistent with the critical role of the cell wall remodeling in cell expansion/elongation, BIN2 was found to phosphorylate and inactivate CESA1 (Cellulose Synthase A1, an important enzyme involved in the primary cell wall biosynthesis [45,46]), thus inhibiting cellulose biosynthesis and cell elongation in dark-grown hypocotyls (Figure 1) [47]. It remains to be investigated if BIN2 and its homologs can interact, phosphorylate, and directly regulate additional cellular components known to be essential for cell expansion/elongation.

The plant GSK3-like kinases also regulate cell division via their phosphorylation activities towards cyclins (Figure 1), similar to what was previously known for the animal GSK3 kinases. The rice OsSK22/GSK2 can phosphorylate a U-type cyclin, CYC U2, to enhance the activity of the CYC U2/CDKA (Cyclin-Dependent Kinase A) complex, leading to accelerated cell division of abaxial sclerenchyma cells of the rice lamina joint to cause the erected leave phenotype [48]. Interestingly, the OsSK22/GSK2-catalyzed phosphorylation of CYC U2 in the rice mesocotyls promotes the interaction of CYC U2 with a rice F-box protein, DWARF3 (D3) critical for strigolactone signaling [49], leading to rapid degradation of CYC U2, reduced cell division, and shorter mesocotyls [50]. The different impact of the rice OsSK22/GSK2 on CYC U2 and cell division might be explained by cell type-specific expression of CDKA and D3. Additional support for the involvement of plant GSK3-like kinase-catalyzed cyclin phosphorylation in regulating cell division was provided by a tobacco study. A tobacco BIN2 ortholog, NbSKη (*Nicotiana benthamiana* Shaggy-like Kinase η), was reported to regulate cell division by phosphorylating NbCycD1;1 (*Nicotiana benthamiana* cyclin D1.1), leading to the 26S proteasome-dependent NbCycD1;1 degradation and reduced cell division [51].

### 3.2. Tissue-Specific Regulation of Stomatal Development

Stomata on the leaf surface are essential for plant growth and survival by regulating the CO_2_ uptake and the release of oxygen and water. It is well established that the stomata development is regulated by a MAPK (Mitogen-Activated Protein Kinase)-mediated signaling cascade. This pathway consists of YODA (YDA, also known as MAPKKK4 for MAPK Kinase Kinase4), four MAPK kinases (MKK4, MKK5, MKK7, and MKK9), two MAPKs (MPK3 and MPK6), and a basic helix-loop-helix (bHLH) transcription factor SPEECHLESS (SPCH) that initiates the stomata differentiation (Figure 1) [52,53]. Earlier studies demonstrated that plant GSK3-like kinases interact with multiple components of the MAPK signaling cascade to influence the stomatal development [54,55,56,57]. It was reported that BIN2 strongly phosphorylates and inhibits YDA and MKK4/MKK5 (MKK4/MKK5 can also be phosphorylated by AtSK11 and AtSK32), resulting in accumulation of SPCH in the nucleus and enhanced stomata formation in dark-grown cotyledons and light-grown leaves (Figure 1) [54,56]. Interestingly, BIN2 can also interact directly with and phosphorylate SPCH to promote the 26S proteasome-mediated degradation of SPCH, thus inhibiting the stomata formation in the hypocotyls [55]. The opposite role of BIN2 in stomatal development might be mediated by tissue-specific interactions of BIN2 and its homologs with the stomata lineage “polarity proteins” that include POLAR (POLAR LOCALIZATION DURING ASSYMMETRIC DIVISION AND REDISTRIBUTION) [58] and BASL (Breaking of Asymmetry in the Stomatal Lineage) [59]. BIN2 is recruited to the cytosol by POLAR and is transiently polarized within the cytosol with BASL, thus attenuating the MAPK signaling cascade and activation of SPCH, while the nuclear-localized BIN2 phosphorylates and negatively regulates SPCH [60]. 

### 3.3. Root Development

The plant roots not only firmly anchor the plants in the soil but also actively acquire essential mineral nutrients and water for optimal plant growth and development, which is largely determined by rooting depth, root hairs, and root branching. A recent study implicated BIN2 in regulating root meristem development [61]. It was shown that BIN2 phosphorylates UPB1 (UPBEAT1), an atypical bHLH transcription factor that was thought to regulate balance between cell division and differentiation in the root meristem [62], to inhibit the root meristem development [61]. An earlier study showed that BIN2 is also involved in the root hair development [63,64] by regulating a root-specific trimeric transcriptional complex composed of a MYB protein WER (WEREWOLF) [65], a bHLH-type transcription factor GLABRA 3 (GL3) or its close homolog EGL3 (ENHANCER OF GLABRA3) [66], and a WD40-repeat protein TTG1 (TRANSPARENT TESTA GLABRA1) [67]. The WER-GL3/EGL3-TTG1 complex promotes expression of a homeobox protein GL2 to repress transcription of root hair-specific genes. In the absence of BRs, the constitutively-active BIN2 phosphorylates EGL3 and inhibits the nuclear localization of EGL3, leading to decreased formation of the WER-GL3/EGL3-TTG1 trimeric complex in the nuclei and suppressed GL2 expression (Figure 1) [63]. BIN2 could also phosphorylate TTG1 to directly inhibit the transcriptional activity of the WER-EGL3-TTG1 complex, thus releasing its inhibitory effect on root hair-specific genes and triggering root hair development [63]. It is interesting to note that an Arabidopsis arabinogalactan peptide known as AGP21 was demonstrated to affect the root hair cell fate via a BIN2-dependent manner, providing additional support for the involvement of BIN2 in the root hair development [68]. In addition, the plant GSK3-like kinases were found to be involved in the lateral root development (Figure 1). It was reported that BIN2 phosphorylates and activates ARF7 (auxin response factor7) and ARF19 by blocking their interactions with AUX/IAA (auxin/indole-3-acetic acid) transcriptional repressors [69], leading to activation of Lateral organ Boundaries-Domain16/Asymmetric Leaves-like 18 (LBD16/ASL18) and/or LBD29/ASL16 known to promote the lateral root formation [70]. 

### 3.4. Vascular Development

In addition to regulating the cell specification of guard cells in leaves and hair cells in roots, BIN2/AtSK21 and its homologs were recently shown to regulate xylem differentiation through their interactions with the TDIF (Tracheary element Differentiation Inhibitory Factor) signaling pathway (Figure 1) [71]. All members of the subgroup I (AtSK11, AtSK12 and AtSK13) and II (AtSK21/BIN2, AtSK22/BIL2 and AtSK23/BIL1) of the Arabidopsis GSK3-like kinase family directly interact with the cell surface-localized TDR (TDIF RECEPTOR, also known as PXY for Phloem intercalated with Xylem [72]). All these Arabidopsis GSK3-like kinases except AtSK23/BIL1 were found to inhibit the xylem differentiation through inactivation of BES1, a key transcription factor of the BR signaling pathway known to promote xylem differentiation (Figure 1) [71,73]. By contrast, AtSK23/BIL1 inhibits the xylem differentiation to maintain the cambial cell identity by regulating ARFs of the auxin signaling (Figure 1). It was shown that AtSK23/BIL1 phosphorylates ARF5 and further increases ARF5’s inhibitory impact on vascular cambial activity through upregulating ARR7 (Arabidopsis Response Regulator 7) and ARR15 [73], which are two negative regulators of cytokinin signaling [74]. BIL1 activity is inhibited by the TDIF-PXY/TDR signaling module, resulting in reduced expression of ARR7 and ARR15 and enhanced vascular cambial activity [73]. In addition to their involvement in xylem differentiation, plant GSK3-like kinases were recently shown to regulate the ratio of sieve elements (SEs) and companion cells (CCs) in the phloem tissue (Figure 1) [75]. Pharmacological inhibition or genetic elimination of GSK3-like kinases resulted in an increased ratio of SE/CC whereas overexpression of a gain-of-function mutant variant *bin2-1* slightly decreased the SE/CC ratio, suggesting that the GSK3-like kinases might function redundantly as a cell-fate switch during the phloem development. 

### 3.5. Reproductive Development

The developmental phase change from vegetative to reproductive growth and the subsequent formation of various floral organs are essential for flowering plants. Plant GSK3-like kinases are likely involved in these processes. In Arabidopsis, AtSK41 is expressed predominantly in inflorescences that contain flowers of varying developmental stages [76], while AtSK11 and AtSK12 are highly expressed in various floral tissues [77]. Importantly, silencing AtSK11/12 increased the number of perianth organs and altered the gynoecium patterning, revealing a functional role in the plant flower development. At least three alfalfa GSK3-like kinase genes and one petunia GSK3-like kinase gene were detected in developing flowers [9,78], but it remained unknown how these GSK3-like kinases regulate plant flowering or flower development. A recent study revealed that the Arabidopsis AtSK12 binds and phosphorylates CO (CONSTANS) at the Thr^119^ residue, a B-box zinc finger transcription factor known to be crucial for the photoperiodic regulation of flowering [79], leading to CO degradation and delayed flowering (Figure 1) [80]. It is important to note that BIN2/AtSK21 and its homologs could indirectly regulate the plant flowering process via its inhibitory effect on BES1/BZR1 known to regulate expression of several key transcriptional factors of the floral transition (Figure 1) (reviewed in [81]). It is also interesting to mention that several earlier studies revealed predominant expression of several members of the subgroup III in anthers and mature pollens [78,82,83], suggesting their potential role in regulating male gametogenesis and pollen development.

In addition, plant GSK3-like kinases are implicated in the seed and fruit development (Figure 1). In Arabidopsis, AtSK11/12 were recently shown to phosphorylate TTG1 (at Ser^215^), which not only regulates the formation of trichome of the leaves and root hairs of the roots but also affects seed development [84], to regulate carbon partitioning between zygotic and maternal seed tissues, leading to increased fatty acid production in the embryo and reduced synthesis of mucilage and flavonoid pigments in the seed coat [85]. It is important to note that the AtSK11/12-catalyzed Ser^215^ phosphorylation of TTG1 had little impact on the development of trichomes or root hairs. In rice, OsSK41/OsGSK5, which is encoded by a quantitative trait locus known as q*TGW3* (quantitative-trait locus for *Thousand-Grain Weight3*), phosphorylates OsARF4 (*Oryza sativa* Auxin Response Factor4) to negatively regulate the grain size and weight [86,87,88]. In addition, OsSK22/GSK2 can phosphorylate a rice transcriptional factor OsGRF4 (growth-regulating factor4 [89,90,91]), to inhibit its transcriptional activity, thus reducing the grain size and yield [92]. A recent genetic study with the Indian dwarf wheat (*Triticum sphaerococcum*, one of the six subspecies of hexaploidy wheat) discovered that single amino acid changes in the conserved TREE (Thr-Arg-Glu-Glu) motif of a wheat GSK3-like kinase encoded by the *Tasg-D1* (*Ta*, *Triticum aestivum*, *sg*, semispherical grain, *D1*, the first *sg* gene identified in genome *D*) locus, are responsible for the unique semispherical grain shape [93]. However, it remains to be investigated how TaSG-D1 works in wheat to regulate the grain shape. The plant GSK3-like kinases might also regulate the fruit ripening process (Figure 1). A recent study showed that overexpression of a grapevine GSK3-like kinase (VvSK7 for *Vitis vinifera* Shaggy-like Kinase7) in tomatoes delayed fruit ripening, which seems to be consistent with the finding of that treatment of grapevines with BRs or GSK3-kinase inhibitor at the veraison stage promoted berry ripening with larger/heavier berries [94]. Interestingly, an earlier study found that another grapevine GSK3-like kinase, VvSK1, was induced by sugars and might be involved in transport and accumulation of sugars during the berry ripening process by regulating expression of several sugar transporters [95]. 

### 3.6. Photomorphogenesis

The discoveries of two photomorphogenesis mutants, *det2* (*de-etiolated2*) and *cpd* (*constitutive photomorphogenesis*), as BR-deficient mutants demonstrated the involvement of BRs in photomorphogenesis that includes reduced hypocotyl elongation, biosynthesis of chlorophylls and anthocyanins, development of chloroplasts and true leaves [96,97]. Given its key regulatory role in BR signaling, BIN2 must have important functions in regulating photomorphogenesis (Figure 1). BR-insensitive gain-of-function *bin2* mutations give rise to constitutive photomorphogenesis phenotypes [19,22], whereas dominant gain-of-function mutations (*bes1-D* and *bzr1-D*) in BES1/BZR1 suppress the de-etiolated phenotypes of dark-grown seedlings of *det2* and *bri1* [25,26]. A recent study showed that the binding of BIN2 with HY5 (long hypocotyl5), a well-studied positive regulator of photomorphogenesis downstream of multiple photoreceptors [98], resulted in a stronger phosphorylation activity of BIN2 towards BZR1 and reduced abundance of BZR1, thus promoting photomorphogenesis [99]. Interestingly, CRY1 (CRYPTOCHROME 1), a blue light receptor, could directly interact with both BIN2 and BZR1 to promote the BIN2-catalyzed BZR1 phosphorylation in a light-dependent manner, leading to inhibition of hypocotyl elongation [100]. While the HY5/CRY1-BIN2 interaction stimulates the BIN2-catalyzed BZR1 phosphorylation to regulate hypocotyl elongation, the interaction of BIN2 with PIF4 (Phytochrome Interacting Factor4), which is a key component of the phytochrome signaling pathway [101], enhances the BIN2-catlyzed PIF4 phosphorylation and reduces PIF4 stability to inhibit the hypocotyl elongation [102]. A recent study also implicated BIN2 in regulating another key aspect of plant photomorphogenesis, light-stimulated development of mature chloroplasts [103]. It was discovered that BIN2 phosphorylates GLK1 (GOLDEN2-LIKE1), a transcription factor important for chloroplast development [104], to increase its protein stability and transcriptional activity, thus promoting chloroplast development.

## 4. Multiple Functions in Plant Stress Response

### 4.1. Abiotic Stress

Beyond their roles in plant growth and development, plant GSK3-like kinases appear to function in plant stress tolerance. As a matter of fact, the role of plant GSK3-like kinases in plant stress response was suggested a few years before the discovery of their involvement in BR signaling. A 1999 study discovered that the Arabidopsis AtGSK1/AtSK22 rescued the salt-hypersensitive phenotype of yeast (*Saccharomyces cerevisiae*) mutants lacking either calcineurin or the yeast GSK3-like kinase and that its transcript abundance was induced by NaCl [105]. Importantly, overexpression of AtGSK1 in Arabidopsis resulted in the constitutive activation of several salt stress-responsive genes and greatly enhanced the plant salt tolerance (Figure 1) [106]. Additional gene expression analyses revealed salt stress-upregulation of three other GSK3-like kinase genes (*AtSK13*, *AtSK31*, and *ATSK42*) in Arabidopsis [105,107]. Importantly, treatment of Arabidopsis plants with bikinin, a known inhibitor of a subset of the plant GSK3-like kinases [12], greatly reduced the salt stress tolerance, providing a crucial pharmacological support for the role of the plant GSK3-like kinases in the salt stress tolerance [108]. A biochemical study suggested that Arabidopsis AtSK11/ASKα enhances the plant salt stress tolerance by directly phosphorylating a cytosolic isoform of glucose-6-phosphate dehydrogenase (G6PD) [109], the first enzyme of the oxidative pentose phosphate pathway important for the plant stress tolerance [110]. It was discovered that ASK11/ASKα phosphorylated G6PD at the highly conserved Thr^467^ residue within the predicted substrate binding pocket, to enhance the G6PD enzyme activity, thus slowing the stress-triggered production of reactive oxygen species and enhancing the salt tolerance (Figure 1) [109].

The role of plant GSK3-like kinases in salt stress is likely evolutionarily conserved in angiosperms. OsSK41/OsGSK5 in rice and its alfalfa orthologue MsK4 (*Medicago sativa* protein Kinase4), were previously shown to enhance salinity tolerance in transgenic rice and Arabidopsis, respectively [111,112]. GmGSK (*Glycine max* GSK), a soybean GSK3-like kinase highly homologous to AtSK11, was induced by various abiotic stresses and could confer the salt/osmotic tolerance to yeast cells [113]. At least two GSK3-like kinases from wheat, TaGSK1 (*Triticum aestivum* GSK3-like Kinase1) and TaSK5 (*Triticum aestivum* Shaggy Kinase5), could confer salt tolerance to Arabidopsis (Figure 1) [114,115]. Interestingly, certain members of the plant GSK3-like kinase family could also inhibit the salt stress tolerance. For example, knockout of OsSK21/OsGSK1, which is the ortholog of the Arabidopsis BIN2/AtSK21, leads to enhanced tolerance to various abiotic stresses, such as drought, heat, salt, and cold [116]. Similarly, silencing of several barley GSK3-like kinase genes stimulated seedling growth under both normal and high salinity conditions [117], whereas overexpression of a potato GSK3-like kinase, StSK21 (*Solanum tuberosum* SK21) in Arabidopsis, resulted in enhanced salt sensitivity [118]. However, little is known how these GSK3-like kinases inhibit the salt tolerance. A recent study suggested that the plasma membrane-recruited BIN2 could directly phosphorylate and inactivate SOS2 (Salt Overly Sensitive2), a protein serine/threonine kinase known to play a critical role in regulating the salt stress response [119,120], thus facilitating recovery growth for salt stressed plants [121].

Drought stress is another important environment factor that greatly reduces plant growth and crop yield. Under severe drought stress, plants have to coordinate growth and stress responses for their survival. Several recent studies have demonstrated crucial roles of the GSK3-like kinases in regulating the plant drought stress response (Figure 1). In Arabidopsis, BIN2 positively mediates drought stress signaling via regulating an Arabidopsis transcription factor RD26 (Responsive to Desiccation26) and DSK2 (DOMINANT SUPPRESSOR of KAR2), a member of the ubiquitin receptor family [122,123]. BIN2 phosphorylates and stabilizes RD26, which binds BES1 to suppress the expression of BES1-target genes involved in plant growth but activates dehydration-responsive genes [122]. BIN2 could also regulate the drought stress response by phosphorylating and activating DSK2, a ubiquitin receptor protein in autophagy known to be involved in the plant stress response [124], which subsequently interacts with BES1 to promote autophagy-mediated BES1 degradation [123]. BIN2 also phosphorylates TINY, an Arabidopsis AP2/ERF (Apetala2/Ethylene Response Factor2)-type transcription factor capable of activating many drought-responsive genes [125], thus stabilizing TINY and enhancing its function in drought tolerance [126]. A similar stimulatory function in drought stress was also discovered for MmSK, a Mulberry (*Morus alba* var. *multicaulis*) shaggy-like protein kinase [127]. It was found that MmSK was induced by various abiotic stresses and transgenic silencing of MmSK in mulberry compromised the drought tolerance accompanied by a marked reduction in the drought-induced accumulation of osmotic regulators. It is well known that mild drought stress enhances root extension into deeper/moist soil, and a recent study suggested that AtSK11/12 plays a negative role in such a drought tolerance mechanism [128]. An *atsk11 atsk12* double mutant exhibited a stronger mild stress-induced root growth stimulation and further study suggested that AtSK11/12 might regulate expression of several extensin genes involved in cell wall reinforcement via an Arabidopsis bHLH protein, bHLH69 [128].

In Arabidopsis, BIN2 is known to phosphorylate and inhibit at least three different transcriptional factors, including BZR1, CESTA, and ICE1 (Inducer of CBF Expression1) [129,130,131], to weaken the plant cold tolerance via their reduced binding activity to the promoters of three redundant CBFs (C-repeat binding factors) known to be key regulators of the plant cold stress response (Figure 1) [132]. It was thought that the BIN2-catalyzed phosphorylation of CESTA, a bHLH protein known to regulate BR signaling [133], reduces its protein stability and transcriptional activity and inhibits its SUMOylation-mediated nuclear localization [134], thus diminishing the plant cold tolerance via both CBF-dependent and CBF-independent mechanisms [131]. BIN2 can also bind and phosphorylate ICE1, to promote the interaction of ICE1 with an E3 ubiquitin kinase HOS1 (high expression of osmotically responsive gene1) [130,135], leading to ICE1 degradation, reduced CBF expression, and compromised cold tolerance. It is interesting to note that PIF4, a known BIN2 substrate that synergistically interacts with BZR1 to regulate growth and stress tolerance [102,136], inhibits the expression of CBFs and their target genes, suggesting a positive role of BIN2 in the plant cold stress response. Further studies are needed to fully understand how BIN2 is regulated to balance growth and cold tolerance.

### 4.2. Biotic Stress

In addition to abiotic stress, plant GSK3-like kinases are also involved in the plant defense responses against pathogenic microbes (Figure 1). For example, CaSK23, a putative GSK3-like kinase in pepper (*Capsicum annuum*), plays important negative roles in the pepper immunity by regulating the salicylic acid, jasmonic acid (JA) and ethylene-dependent pathogenesis-related (PR) proteins [137]. Silencing of CaSK23 increased expression of several well-known immunity-associated marker genes and attenuated susceptibility of the pepper plant to *Ralstonia solanacearum,* a soil-borne bacterial pathogen causing the plant wilting disease in a wide range of crop plants. Similarly, MsK1, a GSK3-like kinase in alfalfa, also negatively regulates the plant immunity. It was shown that the protein abundance and its kinase activity were greatly reduced in response to a pathogenic elicitor and that overexpressing MsK1 in transgenic Arabidopsis plants enhanced susceptibility to *Pseudomonas syringae*, one of the best studied plant pathogens [138]. By contrast, the Arabidopsis AtSK11 was rapidly induced upon activation of several innate immunity receptors, and loss of AtSK11 in Arabidopsis increased susceptibility to *Pseudomonas syringae* [139], revealing a positive role of AtSK11 in the plant innate immunity. 

The plant GSK3-like kinases were also implicated in the symptom development of host plants infected by geminiviruses, which are the largest group of known plant viruses and cause devastating losses of many important crops. Several studies showed that the C4 proteins of some geminiviruses interact with BIN2 and its homologs in several different plant species to induce the characteristic viral symptoms [51,140,141,142,143,144,145,146]. In rice, OsSK22/GSK2 phosphorylates OsJAZ4 (Jasmonate ZIM-domain4) to prevent formation of the OsJAZ4-OsJAZ11 heterodimers and the OsJAZ4-OsNINJA corepressor complex, resulting in proteasome-mediated degradation of OsJAZ4 that negatively regulates JA signaling and the antiviral defense (Figure 1) [147]. Interestingly, OsSK22/GSK2 also interacts with and phosphorylates OsMYC2, a key positive component of the rice JA signaling [148], to promote OsMYC2 degradation and to reduce JA-mediated resistance to rice stripe virus [149]. Further studies are needed to fully understand the opposite effects of different GSK3-like kinases in the plant antiviral response.

In addition to the plant defense responses against microbial pathogens, the plant GSK3-like kinases are also involved in the plant-rhizobium symbiosis. LSK1 and LSK2, two members of the *Lotus japonicus* GSK3/Shaggy-like kinase family, were found to be induced upon infection with its natural symbiotic bacterium. Silencing or elimination of LSK1 resulted in increased nodule formation, revealing a negative role in the symbiotic nodulation of the legume [150]. Consistently, GmSK2-8 and its close homologs of the subgroup II of the soybean (*Glycine max*) GSK3/Shaggy-like kinases, which were upregulated by high salinity, also inhibit nodule formation under salt stress [151]. The soybean GSK3-like kinases likely phosphorylate two highly similar GmNSP1 (*Glycine max* Nodulation Signaling Pathway1) proteins, which are the key transcription factors essential for symbiotic nodulation in soybean [151,152], thus reducing their DNA binding activities and inhibiting rhizobial infection and symbiotic nodulation under salt stress (Figure 1). 

A discussion on the involvement of plant GSK3-like kinases in biotic/abiotic stress is not complete without mentioning their potential roles in the plant wounding response. Both biotic and abiotic stresses can cause physical damages to plants that have evolved a sophisticated wounding response to prevent microbial infections [153]. An earlier study in alfalfa identified a wound-induced/activated GSK3-like kinase known as WIG for Wound-Induced GSK3 [154] (Figure 1). However, it remains to be investigated how such a wound-induced GSK3-like kinase regulates the plant wounding response that involves multiple signaling pathways including the MAPK phosphorylation cascade and JA signaling [153]. 

## 5. Perspective

Since the initial discoveries of plant GSK3-like kinases in the early 1990s, this highly conserved protein Ser/Thr kinase family was demonstrated to have versatile functional roles in a wide range of growth and development processes and environmental responses. However, little is known how the plant GSK3-like kinases execute their physiological functions except for their involvement in a few well-studied signaling pathways. Given redundant/distinctive functions and multiple members of the GSK3-like kinase family in each studied and sequenced plant species, it is anticipated that a large number of protein substrates and interacting proteins of these highly conserved kinases will be identified in the near future. Further investigations are also needed to understand the biochemical mechanisms by which the plant GSK3-like kinases are dynamically and precisely regulated in tissue/cell type-specific manners in response to a wide range of developmental and environmental signals. These future studies will certainly enhance our understanding on how plants balance growth and stress tolerance under various environmental conditions, leading to novel strategies of engineering important crops for higher yields with stronger stress tolerance and plant defense.

## Figures and Tables

**Table 1:** Names, Gene/Protein IDs, and Known Substrates of the Plant GSK3-Like Kinases.

Name(s)	Protein/*Locus* ID	Substrate(s)	
*Arabidopsis thaliana*			
**AtSK11**/ASK1/ASKα	At5g26751	TTG1, G6PD, MKK4, MKK5	
**AtSK12**/ASK3/ASKγ	At3g05840	CO, TTG1	
**AtSK13**/ASK5/ASKε	At5g14640		
**AtSK21**/BIN2/ASK7/ASKη /UCU1/DWF12	At4g18710	BZR1/BES1, BSKs, YDA, MKK4, MKK5, SPCH, UPB1, TTG1, EGL3, ARF7, ARF19, RD26, TINY, DSK2, CESTA, ICE1, PIF4, GLK1	
**AtSK22**/BIL2/ASK9/ASKι/AtGSK1	At1g06390	BZR1/BES1, BSKs	
**AtSK23**/BIL1/ASK6/ASKζ	At2g30980	BZR1/BES1, ARF5	
**AtSK31**/ASK2/ASKβ	At3g61160		
**AtSK32**/ASK8/ASKθ	At4g00720	MKK4, MKK5	
**AtSK41**/ASK10/ASKκ/AtK-1	At1g09840		
**AtSK42**/ASK4/ASKδ	At1g57870		
*Oryza sativa*			
**OsSK11**/OsGSK2/OSKγ	Os01g14860		
**OsSK12**/OsGSK3	Os01g19150		
**OsSK13**/OsGSK6/GSK1	Os05g04340		
**OsSK21**/OsGSK1/OSKζ	Os01g10840	OsBZR1, LIC	
**OsSK22**/OsGSK7/GSK2	Os05g11730	OsBZR1, DLT, OFP8, OFP3, OsPUB24, CYCU2, OsMYC2, OsGRF4, OsJAZ4	
**OsSK23**/OsGSK4/GSK3	Os02g14130	OsBZR1	
**OsSK24**/OsGSK8/GSK4/OSKη/SKη	Os06g35530	OsBZR1, LIC	
**OsSK31**/OsGSK9	Os10g37740		
**OsSK41**/OsGSK5	Os03g62500	OsARF4	
Other plant species			
**Name(s)**	**Protein/*Locus* ID**	**Species**	**Substrate**
CaSK23	XP_016580896	*Capsicum annuum*	
GmGSK	Glyma.10G144600	*Glycine max*	
GmSK2-8	Glyma.12G212000	*Glycine max*	GmNSP1
LSK1	BAD95891.1	*Lotus japonicus*	
LSK2	BAD95892.1	*Lotus japonicus*	
WIG	*AJ295939*	*Medicago sativa*	
MsK1	*X68411*	*Medicago sativa*	
MsK4	AF432225.1	*Medicago sativa*	
MmSK	ASR74828	*Morus alba var. multicaulis*	
NbSKη	*Niben101Scf12866g00026.1*	*Nicotiana benthamiana*	NbCycD1;1
PeGSK1	*PH01001237G0340*	*Phyllostachys edulis*	PeBZR1
StSK21	*PGSC0003DMG400028428*	*Solanum tuberosum*	
TaGSK1	AAM77397.1	*Triticum aestivum*	
TaSK5	BAF36565.1	*Triticum aestivum*	
TaSG-D1	AGJ93554.1	*Triticum aestivum*	
VvSK1/VvSKθ	XP_002272112	*Vitis vinifera*	
VvSK7/VvSKκ	XP_010657975	*Vitis vinifera*	

## Data Availability

Not applicable.
